# Targeted disruption of influenza A virus hemagglutinin in genetically modified mice reduces viral replication and improves disease outcome

**DOI:** 10.1038/srep23746

**Published:** 2016-04-01

**Authors:** Song Wang, Chao Chen, Zhou Yang, Xiaojuan Chi, Jing Zhang, Ji-Long Chen

**Affiliations:** 1 College of Animal Sciences, Fujian Agriculture and Forestry University, Fuzhou 350002, Fujian, China; 2 Institute of Microbiology, Chinese Academy of Sciences (CAS), Beijing 100101, China

## Abstract

Influenza A virus can cause acute respiratory infection in animals and humans around the globe, and is still a major threat to animal husbandry and public health. Due to antigenic drift and antigenic shift of the virus, development of novel anti-influenza strategies has become an urgent task. Here we generated transgenic (TG) mice stably expressing a short-hairpin RNA specifically targeting hemagglutinin (HA) of influenza A virus, and investigated the susceptibility of the mice to influenza virus infection. We found that HA expression was dramatically disrupted in TG mice infected with WSN or PR8 virus. Importantly, the animals showed reduced virus production in lungs, slower weight loss, attenuated acute organ injury and consequently increased survival rates as compared to wild type (WT) mice after the viral infection. Moreover, TG mice exhibited a normal level of white blood cells following the virus infection, whereas the number of these cells was significantly decreased in WT mice with same challenge. Together, these experiments demonstrate that the TG mice are less permissive for influenza virus replication, and suggest that shRNA-based efficient disruption of viral gene expression in animals may be a useful strategy for prevention and control of a viral zoonosis.

Influenza viruses are negative-sense, single-stranded, segmented RNA viruses, and can be categorized into three types: A, B, and C^1^. Influenza A viruses are further divided into subtypes based on surface proteins called hemagglutinin (HA) and neuraminidase (NA). There are 16 known HA and 9 known NA subtypes^2^. The diversity of influenza A viruses make them have multiple hosts including humans and various animals, such as pigs, chickens, quail, ducks, and turkeys. Influenza A virus can cause highly pathogenic respiratory diseases in animals and humans, leading to enormous morbidity and economic loss annually in the world. In addition, the viruses undergo gradual, continuous change (antigenic drift) and dramatic, abrupt change (antigenic shift), which result in broad epidemic and occasionally pandemic occurrence^3^,^4^,^5^.

To control influenza epidemic and pandemic, various vaccines and antiviral drugs have been developed and recognized for their role in mitigating the health impact of the viral infection^6^. However, despite intensive efforts, there are still restrictions for both strategies to prevent and control influenza A virus^7^,^8^. The presence of frequent mutation and occasional reassortment significantly increases the difficulty in control of these viruses. For example, antibodies produced to influenza A virus as a result of infection or vaccination with earlier strains may not be protective against viruses circulating in later years. These necessitate frequent updating of influenza vaccine components to ensure that the vaccine is matched to circulating viruses. However, antigenic alterations of the virus due to evolutionary shift and drift are notoriously unpredictable. Thus, vaccines are unable to provide immediate protection against outbreaks of unexpected influenza virus strains^9^. The protective capability of currently available influenza vaccines is therefore substantially limited.

Development of antiviral drugs with activity against influenza A viruses is another important strategy in the control of the viral infection. Several antiviral drugs in two classes are currently approved for clinical use: the adamantanes-amantadine and rimantadine, and the neuraminidase inhibitors-oseltamivir and zanamivir^10^,^11^. However, only drugs in second class such as oseltamivir and zanamivir are currently recommended for clinical rational use due to high levels of influenza virus resistance to adamantanes among circulating influenza A virus strains^12^,^13^. Dismayingly, more and more studies revealed that resistance of influenza viruses to oseltamivir and zanamivir is being enhanced^14^,^15^,^16^,^17^. Therefore, novel strategies are needed for preventing ongoing influenza infection.

RNA interference (RNAi) is a process by which double-stranded RNA directs selective silencing of genes with homology to the double strand^18^. Small interfering RNAs (siRNAs) containing 21–26 nucleotide (nt) are mediators of RNAi^19^. Numerous studies have shown that siRNA can significantly suppress viral gene expression and thus protect the host from their infection when delivered into cells *in vitro* or mice *in vivo*^20^,^21^,^22^,^23^,^24^,^25^,^26^,^27^. Importantly, there is compelling evidence indicating that RNAi is critical in curtailing influenza virus infection, which implies a promising approach to treat infection by influenza A virus^28^,^29^. However, this approach has currently limited applications because it can not create a long-term silence of viral genes *in vivo*^30^.

In this study, we constructed an expression vector capable of stably expressing short hairpin RNA (shRNA) that specifically targets conserved sequences of influenza A virus HA, a critical component for the viral infection and replication. The vector was then integrated into mouse genome, which generated the genetically modified mice that stably express the specific shRNA. We have found that HA expression was dramatically disrupted in the transgenic (TG) mice after influenza A virus infection. Furthermore, the resulting progeny of TG mice exhibited stable capacity to reduce influenza A virus replication and disease severity. These results establish an effective strategy for prophylaxis of influenza virus infection. This approach overcomes many of the shortcomings previously experienced, and may be applicable to farm animals that are hosts of influenza virus.

## Results

### Construction of shRNA-based efficient disruption of influenza A virus HA expression *in vitro*

**Figure 1:**
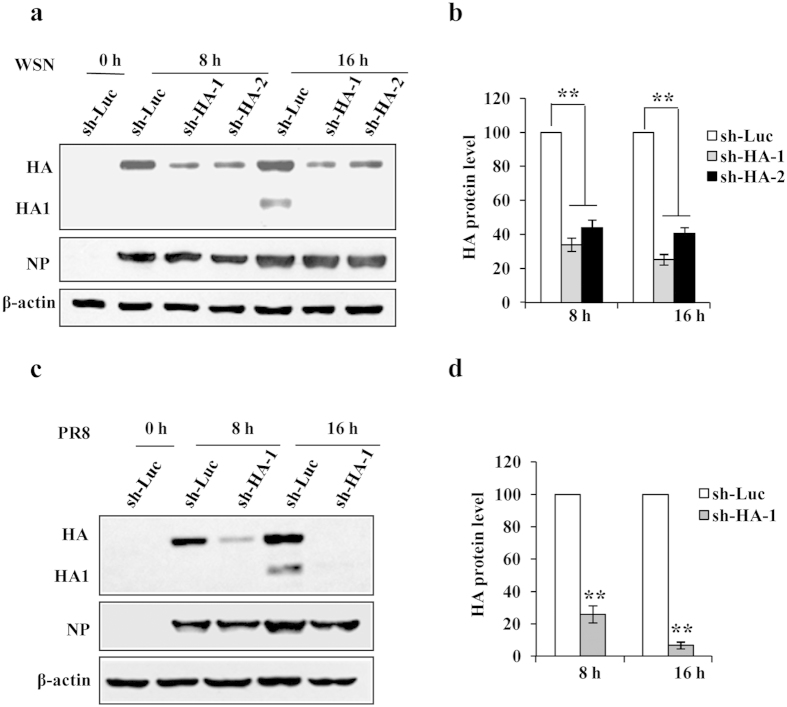
shRNA-based efficient disruption of influenza A virus HA expression in A549 cells. (**a**) A549 cells stably expressing shRNAs targeting either HA (sh-HA-1 and sh-HA-2) or luciferase control (sh-Luc) were infected with WSN virus (MOI = 1) and harvested at the indicated times, followed by Western blotting using the indicated antibodies. These data are representative of three independent experiments with similar results. (**b**) The HA levels shown in panel (**a**) were quantitated by densitometry and normalized to β-actin levels. In each experiment, the HA level in A549 cells expressing sh-Luc is 100. Plotted are the average levels from three independent experiments. **P = 0.001275, sh-Luc *vs.* sh-HA-1 at 8 h; **P = 0.003938, sh-Luc *vs.* sh-HA-2 at 8 h; **P = 0.001094, sh-Luc *vs.* sh-HA-1 at 16 h; **P = 0.002148, sh-Luc *vs.* sh-HA-2 at 16 h. (**c**) A549 cells stably expressing sh-HA-1 or sh-Luc were infected with PR8 virus (MOI = 1) and harvested at the indicated times, followed by Western blotting with the indicated antibodies. Shown are representative immunoblots from three independent experiments with similar results. (**d**) The HA levels shown in panel (**c**) were quantitated as described in (**b**). In each experiment, the HA level in A549 cells expressing sh-Luc is 100. **P = 0.002921, sh-Luc *vs.* sh-HA-1 at 8 h; **P = 0.000326, sh-Luc *vs.* sh-HA-1 at 16 h.

Influenza A virus contains a segmented RNA genome. Two of the eight RNA segments encode the major surface glycoproteins: HA and NA. HA is responsible for virus attachment to the receptor of host cells to initiate an infection and entry of the viral genome into the target cell, which are critical for virus replication circle^31^. Thus, we selected HA as a target gene. To effectively silence different subtypes of HA, we sought to design shRNAs targeting conserved sequences of influenza A virus HA. As shown in Supplementary Tables S1 and S2, although there are variations in the highlighted sequences among HA subtypes, they are highly conserved in H1 and H5 subtypes. Therefore, these sequences were selected as targets of shRNAs. Two shRNAs were synthesized and cloned into pSIH-H1-GFP vector respectively. To test the interference efficiency, A549 cell lines stably expressing shRNAs targeting HA (sh-HA-1 or sh-HA-2) or luciferase control (sh-Luc) were generated. These cell lines were then infected with WSN virus and harvested at 8h and 16h post infection, followed by analysis of Western blot. We found that A549 cells expressing either HA-specific shRNA exhibited a significantly reduced HA expression as compared with that of control cells. Remarkably, sh-HA-1 had better interference efficiency than sh-HA-2 (Fig. 1a,b). To further determine the effect of sh-HA-1 on HA expression, the cell lines were infected with PR8 virus. Similarly, expression of PR8 virus HA was dramatically disrupted by the shRNA (Fig. 1c,d). We also found that viral NP expression was clearly reduced in A549 cells expressing HA-specific shRNA when they were infected with low multiplicity of infection (MOI) of the virus, while NP levels were only slightly decreased when infected with high virus MOI (Fig. S1 and Fig. 1a). These experiments demonstrated that shRNA sh-HA-1 could effectively silence both WSN HA and PR8 HA *in vitro*, and thus was used in further studies.

### Generation of TG mice stably expressing shRNA that effectively silences HA of influenza A virus

**Figure 2:**
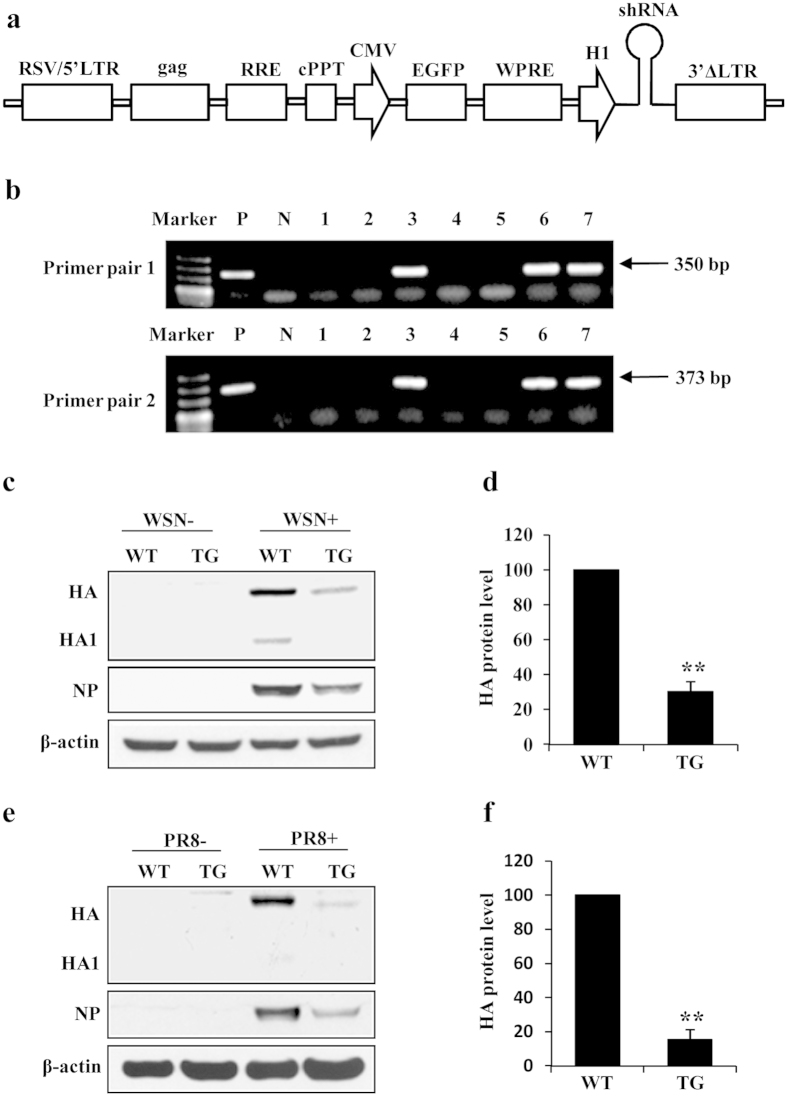
Transgenic (TG) mice expressing shRNA specific for HA can remarkably inhibit HA expression *in vivo*. (**a**) Schematic diagram of shRNA expression vector (pSIH-H1-GFP) used in this study. Transcription of shRNA is driven by the H1 promoter. EGFP expression is driven by the cytomegalovirus (CMV) promoter. RSV/5′LTR, gag, RRE, cPPT, WPRE and 3′ΔLTR are lentivirus components. (**b**) The TG mice expressing shRNA targeting HA were genotyped by PCR using mouse tail DNA as a template and two primer pairs. Shown is representative genotyping of TG and wild type (WT) mice. Numbers 1–7, representative TG mice and WT littermates; P, positive control; N, negative control. (**c**) WT and TG mice intranasally infected with WSN virus for 4 days were sacrificed, and the lungs were homogenized, followed by Western blotting with the indicated antibodies. Shown are representative immunoblots from three experiments with similar results. (**d**) The HA levels shown in panel (**c**) were quantitated by densitometry and normalized to β-actin levels. In each experiment, the HA level in WSN infected WT mice is 100. **P = 0.003894. (**e**) WT and TG mice infected with PR8 virus were treated as described in (**c**). Western blotting was performed using the indicated antibodies. The results are representative of three independent experiments. (**f**) The HA levels shown in panel (**e**) were quantitated as described in (**d**). In each experiment, the HA level in PR8 infected WT mice is 100. **P = 0.002828.

Since the shRNA sh-HA-1 could deplete HA of influenza A virus *in vitro*, we next generated TG mice using shRNA expression vector that carries sh-HA-1 sequences located downstream of an H1 promoter (Fig. 2a). Three lines of transgenic BALB/c mice that express the specific shRNA were developed. The progeny of these TG mice were genotyped by the PCR with 2 pairs of primers, which exhibited different bands of 350 bp (Primer pair 1) and 373 bp (Primer pair 2) (Fig. 2b). Statistical analysis showed that the ratio of positive generations were about 50%, which was consistent with the expected Mendelian frequency. To test the interference efficiency of shRNA targeting influenza A virus HA, six week-old TG mice and WT littermates were intranasally infected by WSN virus or PR8 virus. Then the viral HA expression in lungs were examined by Western blotting. Consistent with the *in vitro* studies, HA expression was markedly knocked down in TG mice as compared with that of WT mice after infection with either WSN virus (Fig. 2c,d) or PR8 virus (Fig. 2e,f). These experiments provide strong evidence that the expression of influenza A virus HA can be effectively disrupted in the genetically modified mice.

### Targeted disruption of influenza virus HA in TG mice causes significantly enhanced resistance of the animals to the virus infection

**Figure 3:**
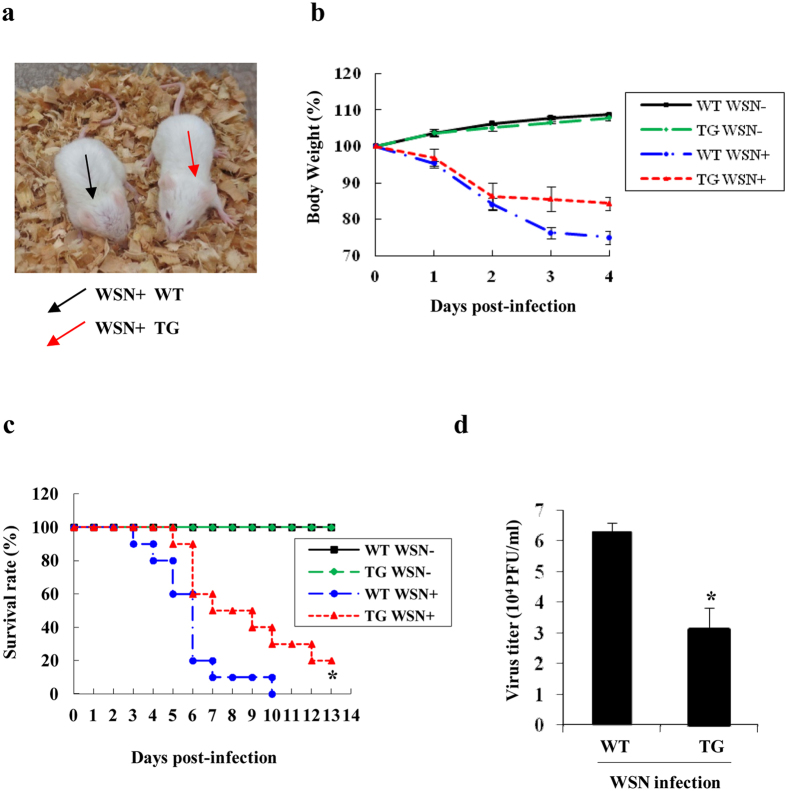
TG mice are resistant to infection with WSN virus as compared to WT mice. (**a**) WT and TG mice were intranasally infected with WSN virus (5 × 10^4^ PFU/mouse) for 4 days. Shown is a representative photograph of the general appearance of the virus infected mice. (**b**) Shown is the body weight changes of WT and TG mice mock infected or infected intranasally with WSN virus. The results are shown as mean percentage weight changes from three independent experiments. (**c**) Survival rate of WT mice (n = 15) and TG mice (n = 15) infected intranasally with WSN virus. Mice were monitored for up to 13 days. During this period, mice were sacrificed when they displayed severe unrelieved distress, hind limb paralysis or excessive weight loss (25% weight loss from initial body weight). Survival curves were compared using a log-rank test (GraphPad Prism 5). *P =  0.0225, WSN infected WT mice *vs.* WSN infected TG mice. (**d**) Viral loads in the lungs of WT and TG mice infected with WSN virus for 4 days were measured by plaque assay. *P = 0.015885.

Since the generated TG mice could significantly decrease the viral HA expression after the infection, we next determined whether silencing HA by the shRNA could protect the TG mice against challenge with influenza A virus. To this end, TG mice and WT littermates were infected with WSN virus, and their resistance to the virus was compared. Interestingly, we found that WT mice infected with WSN virus had severe flu symptoms, as evidenced by reduced activity, ruffled fur and difficult breathing, whereas only mild flu symptoms were observed in TG mice (Fig. 3a). In addition, WT mice displayed a faster body weight loss than TG littermates during the viral infection (Fig. 3b). Moreover, the survival rate of infected TG mice was increased as compared to WT animals. As shown in Fig. 3c, all WT mice were died within 10 days after infection, whereas approximately 33% of TG littermates remained alive, and about 20% of the TG mice finally survived. Statistical analysis showed that TG mice survived significantly longer than WT mice after WSN infection (P < 0.05). Furthermore, viral loads were evaluated in the lungs of the infected mice. As expected, the viral titers in lungs of TG mice were significantly reduced as compared to that of WT mice (Fig. 3d and Fig. S2).

**Figure 4:**
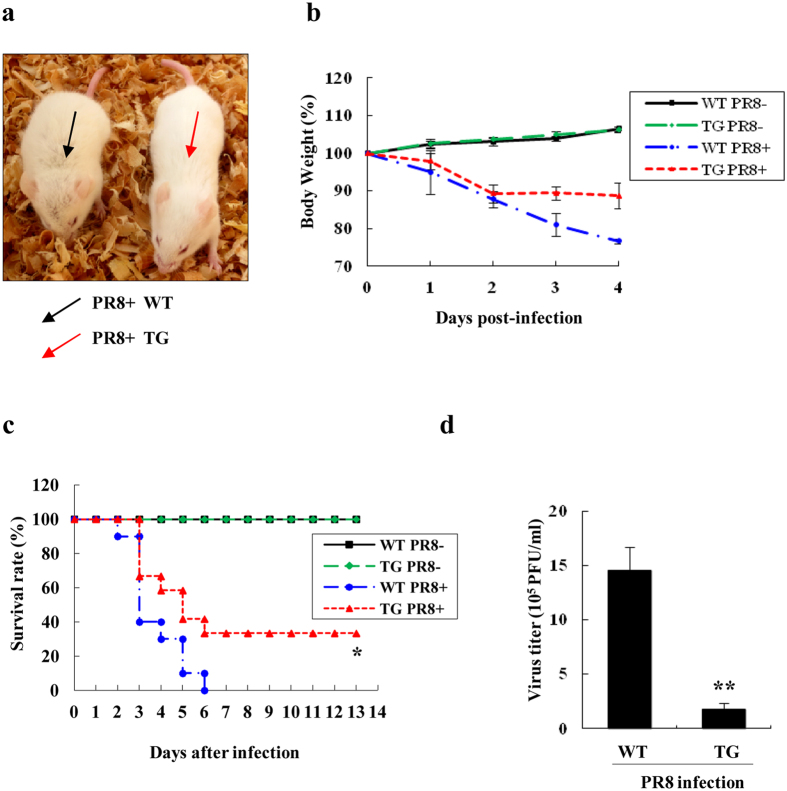
TG mice display resistant to PR8 virus infection compared with WT mice. (**a**) WT and TG mice were intranasally infected with PR8 virus as described in Fig. 3a. Shown is a representative photograph of the general appearance of the virus infected mice. (**b**) Shown is the body weight changes of WT and TG mice mock infected or infected intranasally with PR8 virus. The results are shown as mean percentage weight changes from three independent experiments. (**c**) Survival rate of WT mice (n = 15) and TG mice (n = 15) infected intranasally with PR8 virus was tested as described in Fig. 3c. Survival curves were compared using a log-rank test (GraphPad Prism 5). *P =  0.0356, PR8 infected WT mice *vs.* PR8 infected TG mice. (**d**) Viral loads in the lungs of WT and TG mice infected with PR8 virus were measured as described in Fig. 3d. **P = 0.007122.

To further confirm that disruption of HA expression in TG mice has significant effect on susceptibility of the animals to infection with influenza virus, we used PR8 virus to repeat the experiments presented above. Consistent with the observations in experiments using WSN virus, TG mice showed enhanced resistance to PR8 virus infection, as indicated by mild flu symptoms (Fig. 4a), lower weight loss (Fig. 4b), increased survival rate (Fig. 4c), and significantly reduced viral loads in lungs as compared with those of WT littermates under same experimental condition (Fig. 4d). Together, these data indicate that targeted disruption of the HA in the animals provides a protection against lethal infection with the H1N1 influenza virus.

### Targeted disruption of influenza virus HA in TG mice causes attenuated acute organ injury and reduced pathological changes of organs during the viral infection

**Figure 5:**
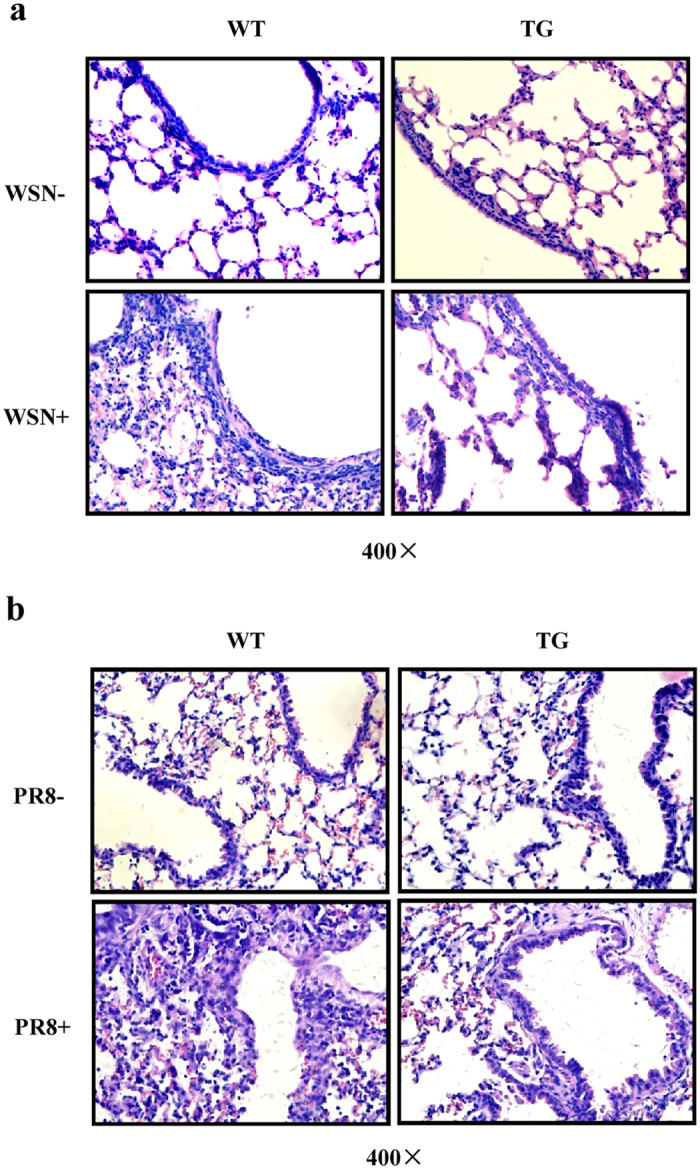
The pathological changes in lungs of influenza virus infected WT and TG mice. (**a,b**) WT and TG mice were intranasally infected with WSN (**a**) or PR8 (**b**) virus. Shown are representative micrographs (magnification, ×400) of the mouse lungs stained with hematoxylin and eosin (HE).

Previous studies have suggested that destructive effects of highly virulent influenza A virus on host organs may be one crucial factor that contributes to the fatal diseases in mammals^32^. To further verify the protective efficacy of silencing HA in TG mice against influenza virus, the pathological changes of mouse organs were therefore examined after infection with WSN or PR8 virus. Strikingly, infection of mice with influenza A virus resulted in acute organ injury, including severe lung lesions and severe spleen and thymus atrophy observed in WT mice, whereas these changes were mild in TG mice (Fig. S3). Moreover, haematoxylin and eosin (HE) staining of the lungs showed that abundant inflammatory cells were present in the alveoli and diffuse in the peribronchiolar and perivascular regions in the lungs of infected WT mice, whereas only a small number of inflammatory cells were seen in the alveoli of TG mice after same infection (Fig. 5). Spleens of infected WT mice showed a dramatic decrease in lymphoid nodule size of white pulps, while this pathological change in infected TG mice was mild (Fig. S4a,b). Likewise, there was a more severe cortical atrophy in the thymus of WT mice than that of TG mice (Fig. S4c,d), which was likely due to a marked loss of lymphocytes during the viral infection^33^.

**Figure 6:**
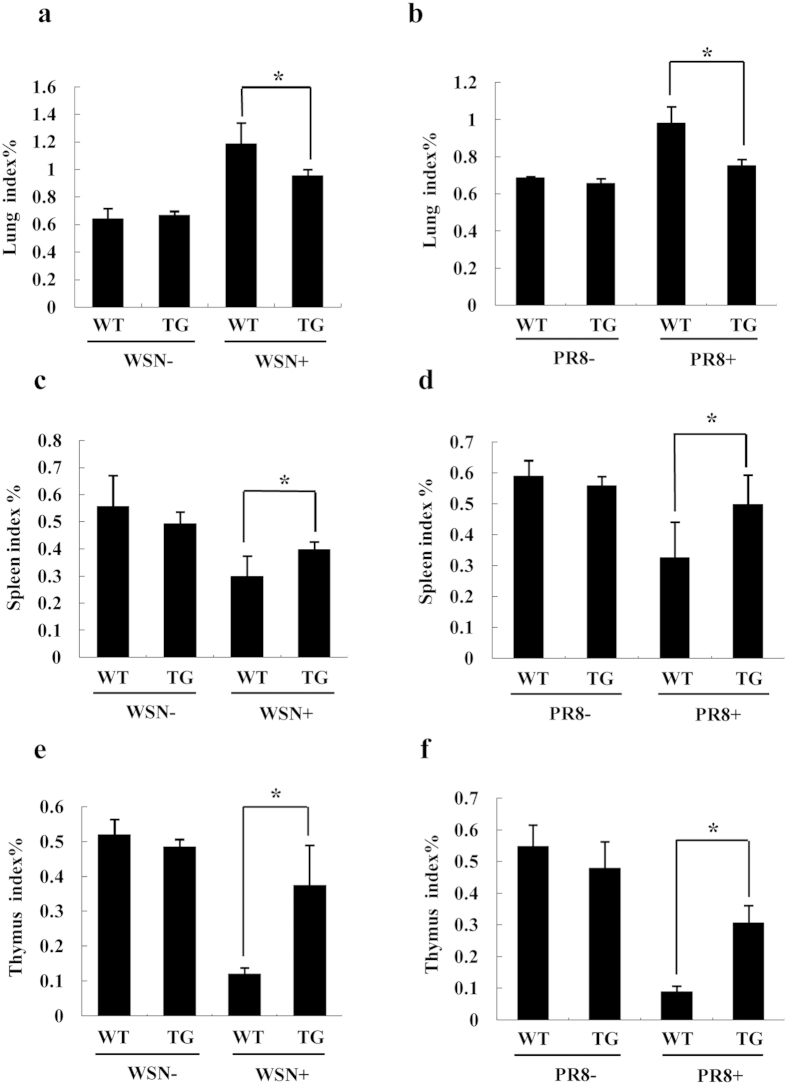
The lung, spleen and thymus index of WT and TG mice infected with influenza virus. (**a,b**) WT and TG mice were infected with WSN (**a**) or PR8 (**b**) virus (5 × 10^4^ PFU/mouse) for 4 days. Then lung index was calculated. Lung index = lung weight (g)/body weight (g) × 100%. (**a**) *P = 0.032695; (**b**) *P = 0.017735. (**c**,**d**) WT and TG mice were infected with WSN (**c**) or PR8 (**d**) virus (5 × 10^4^ PFU/mouse) as described in (**a**,**b**). Then spleen index was calculated. Spleen index = spleen weight (g)/body weight (g) × 100%. (**c**) *P = 0.021756; (**d**) *P = 0.020536. (**e**,**f**) WT and TG mice were infected with WSN (**e**) or PR8 (**f**) virus (5 × 10^4^ PFU/mouse) as described in (**a**,**b**). Then thymus index was calculated. Thymus index = thymus weight (g)/body weight (g) × 100%. (**e**) *P = 0.037937; (**f**) *P = 0.013413.

Because previous studies have shown that low lung index, high spleen index and thymus index correlated well with strong protection against the virus infection^34^, we next evaluated the lung index, spleen index and thymus index. As shown in Fig. 6, TG mice exhibited obviously lower lung index (Fig. 6a,b), higher spleen index (Fig. 6c,d) and thymus index (Fig. 6e,f) than WT mice following a challenge with either WSN virus or PR8 virus. These data demonstrate that silencing influenza virus HA in genetically modified mice leads to attenuated acute organ injury and reduced their pathological changes during the viral infection.

### TG mice can maintain normal levels of white blood cells after challenge with influenza A virus

**Figure 7:**
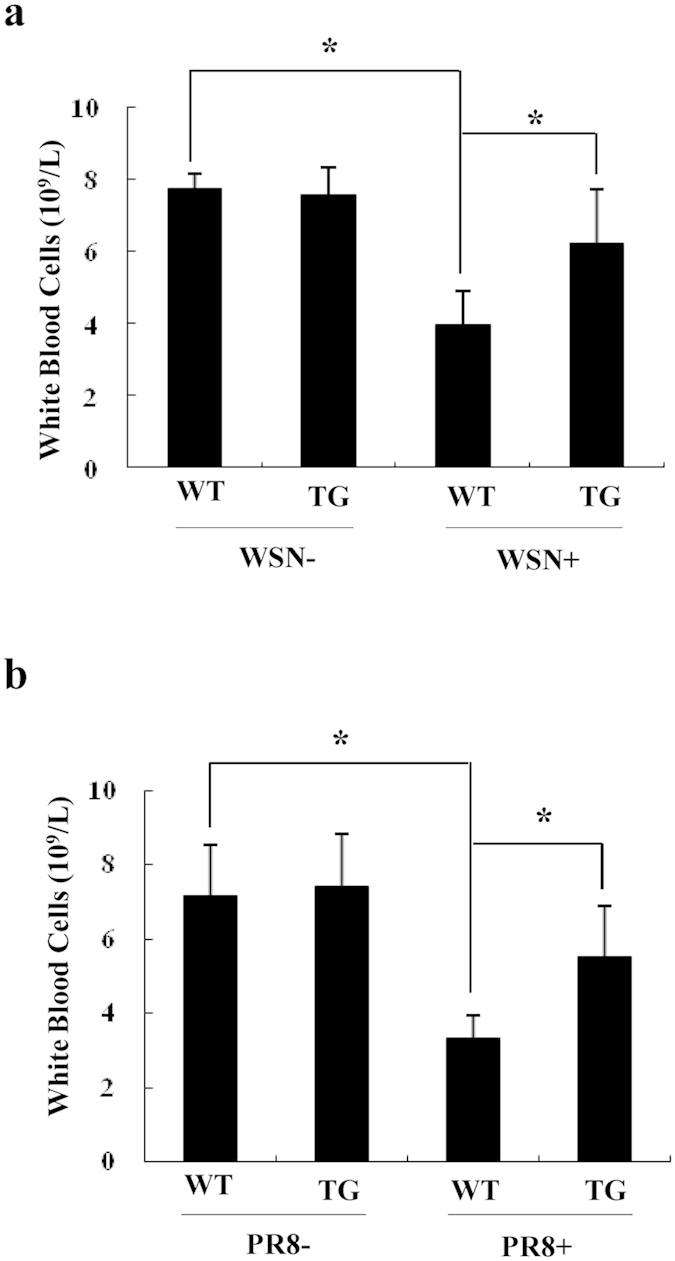
TG mice maintain normal levels of white blood cells after influenza virus infection. (**a**,**b**) WT and TG mice were infected with WSN (**a**) or PR8 (**b**) virus (5 × 10^4^ PFU/mouse) for 4 days. Then mice were sacrificed, and number of white blood cells in mouse peripheral blood was analyzed by blood routine examination. (**a**) *P = 0.019339, uninfected WT mice *vs.* infected WT mice; *P = 0.044106, infected WT mice *vs.* infected TG mice. (**b**) *P = 0.020983, uninfected WT mice *vs.* infected WT mice; *P = 0.044035, infected WT mice *vs.* infected TG mice.

The innate and adaptive immune responses to viral infection mediated by immune cells are rapid and specific, resulting in viral clearance and establishment of immune memory^35^,^36^. To circumvent the host immunity, influenza A viruses have evolved multiple strategies, such as inducing a severe inflammatory response with immune-related complications including significantly reduced numbers of immune cells^32^,^33^. Therefore, we determined whether stable expression of shRNA targeting influenza virus HA in mice had any effect on the numbers of white blood cells in mouse peripheral blood after the virus infection. Indeed, we observed that infection with either WSN virus or PR8 virus resulted in a significant decrease in white blood cells of WT mice (Fig. 7). However, white blood cells in TG mice were close to the normal level after same challenge with the viruses (Fig. 7). Taken together, these results reveal that targeted silence of influenza virus HA in genetically modified mice can effectively mitigate the reduction of white blood cells normally occurring during viral infection.

## Discussion

Influenza virus is still considered to be a global threat and will continue to pose challenges to public health because of its easy transmission, antigenic shift and drift, and the limited efficacy of current vaccines and antiviral drugs^37^. Therefore, development of novel anti-influenza strategies is critically required for counteracting the emergence of highly virulent influenza A virus. Previous studies have shown that shRNA expressed in transgenic chickens can function as a decoy, inhibit influenza virus polymerase and hence interfere with virus propagation^38^. Other experiments have also demonstrated that influenza viral mRNA is direct target of siRNA-mediated interference, and viral genomic RNA (vRNA) and complementary RNA (cRNA) accumulation can be inhibited indirectly^22^. In this study, we generated TG mice stably expressing shRNA that causes the animals reduced susceptibility to influenza virus infection through disrupting expression of viral HA. Moreover, we also determined ability of the genetically engineered mice to pass influenza virus to other mice. We observed that mice housed with infected WT mice showed clearly loss of body weight, while the mice housed with infected TG mice showed an increase of body weight during 5 days housing (Fig. S5), suggesting a reduced ability of TG mice in transmission of influenza virus. This property could have a major impact on susceptibility and propagation of infection at the flock level, even though the survival rate of mice primarily challenged with a high dose of influenza virus was less than 40%. Importantly, the three lines of TG mice in our study exhibited similar capacity to reduce influenza A virus replication and disease severity. The results presented above are representative of those obtained from three lines. Together, these findings have significant implications that genetic modification resistant to influenza virus infection can be developed not only in avian, but also in mammals.

Previous studies have raised some concerns on the safety of genetic modification^39^,^40^,^41^,^42^,^43^. For example, adverse effects derived from RNA interference were observed *in vivo*, such as progressive weight loss, motor dysfunction and animal demise^40^. Therefore, it is important to examine any potential hazards derived from genetic modification. In this study, at least three founder lines of the TG mice expressing shRNA targeting influenza virus HA have been generated. There are no apparent ill-effects on uninfected transgenic animals as compared with the WT littermates. All these mice developed normally and phenotypically normal and fertile without affecting other genetic properties of these lines, even though they have been maintained on a BALB/c background for more than 3 years. In addition, HE staining showed that there was no significant histological difference between WT and TG mice without virus infection (Fig. S2). One possible explanation is that the designed shRNA targeting HA in this study did not share identity with any host genes and the genomic location of the shRNA integrated into mouse chromosome was unlikely to present a risk to the host. However, whether the transgene has any effect on susceptibility of the TG animals to other pathogens requires further studies.

Influenza virus HA, the principal antigen on the viral surface, is the primary target for neutralizing antibodies and is responsible for viral binding to host receptors, and subsequent membrane fusion^44^,^45^. Now it is generally accepted that HA of human influenza viruses prefer to bind to α2-6 sialic acids and HA of avian influenza viruses prefer to bind to α2-3 sialic acids^46^,^47^. The α2-6 sialic acids and α2-3 sialic acids receptors are dominantly expressed in humans and in avian respectively^48^, while both of the receptors are reported to be present in pigs^49^. Based on this, pigs are often considered as mixing vessels for the recombination of human and avian influenza viruses, providing the conditions for gene recombination of influenza viruses to create a new subtype^50^. Therefore, if we could use the technology to develop transgenic pigs that have decreased susceptibility to influenza virus infection, the ability of influenza viruses to cross host species barrier from avian to humans can likely be inhibited to a certain extent. However, this issue remains to be further addressed.

Our approach is also technically applicable to other farm animals that are hosts of influenza A virus, such as chickens, quail, ducks, and turkeys. This approach causes TG animals reduced susceptibility to influenza virus infection and no adverse effects are seen in the host. Even though whether the produced food derived from transgenic farm animals is safe to consumers is still a controversial topic, the application of this technology will very likely bring benefits for the global breeding industry as well as public health, including prevention and control of viral zoonosis. In addition, genetically modified animals generated by using our approach would be useful models for studies of virus-host interaction. In particular, our approach is applicable to generation of animal models for determining function of key viral genes *in vivo* and understanding mechanisms underlying pathogenesis of threatening viruses.

## Materials and Methods

### Ethics statement

The animal protocol used in this study was approved by “the Regulation of College of Animal Sciences, Fujian Agriculture and Forestry University of Research Ethics Committee” (Permit Number PZCASFAFU2014001). All mouse experiments were carried out according to the Regulations for the Administration of Affairs Concerning Experimental Animals approved by the State Council of People’s Republic of China.

### Virus and Antibodies

Influenza virus strains A/WSN/33 (H1N1) and A/PR/8/34 (H1N1) were generated and propagated in specific-pathogen-free (SPF) chicken embryo as previously described^51^. The following antibodies were used in this study: anti-β-actin (Abcam, Cambridge, UK), anti-influenza A virus HA was kindly provided by Dr George F. Gao (Institute of Microbiology, CAS), and anti-influenza A virus NP was obtained as previously described^51^,^52^.

### Cell culture and virus infection

293T, A549 and MDCK cell lines were purchased from American Type Culture Collection (Manassas, VA, USA). Cells were grown in Dulbecco’s modified Eagle’s medium (DMEM) supplemented with 10% fetal bovine serum (FBS), 100 U/mL penicillin and 100 μg/ml streptomycin. For virus infection, cells were washed with phosphate-buffered saline (PBS) and infected with influenza virus at the indicated multiplicity of infection (MOI). After adsorption for 1 h at 37 °C, the cells were washed with PBS and cultured in DMEM containing 2 μg/ml trypsin. *In vitro* cell infection experiments with WSN or PR8 viruses were performed under biosafety level 2 (BSL-2) laboratory conditions.

### Cell extracts and Western blotting

Cell extracts were lysed in RIPA lysis buffer (Cell Signaling Technology; Beverly, MA, USA), according to the manufacturer’s protocols. After adding 2 × loading buffer, lysates were boiled for 5 min. Western blotting was performed as previously described^51^,^52^. Briefly, samples were separated by SDS-polyacrylamide gel electrophoresis, transferred onto a nitrocellulose membrane, and probed with antibodies as indicated. Where indicated, immunoblotting signals were quantified by densitometry.

### RNA interference and generation of stable cell lines

Short hairpin RNA (shRNA)-based knockdown cell lines were generated as described previously^53^. The sequences used in the shRNAs targeting HA genes were as follows: HA shRNA-1: 5′- GGGAGGATGAACTATTACT-3′ and HA shRNA-2: 5′-CATGGAAAGTGTAAGAAAT-3′, and luciferase control shRNA as previously described^54^.

### Generation of HA-knockdown transgenic mice

HA-knockdown transgenic mice were generated by the microinjection method as previously described^55^. The sequences used in the shRNA targeting HA were cloned into an expression plasmid under the H1 promoter. Then the plasmid was linearized by single enzyme digestion of Sca I, separated on 1% agarose gel in 1 × TAE, and purified using the Qiaquick gel extraction kit (Qiagen). The resulting DNA preparations were injected into the pronucleus of fertilized zygotes harvested from BALB/c mice. Genotyping of transgenic mice was performed by the PCR using two pairs of primers: primer 1 forward, 5′- AAATCCTGGTTGCTGTCTCTTTATG-3′, primer 1 reverse, 5′-GGAAGGTCCGCTGGATTGA-3′; primer 2 forward, CGTCCAGGAGCGCACCATCTTCTT, primer 2 reverse, ATCGCGCTTCTCGTTGGGGTCTTT. The HA-knockdown transgenic mice were bred in SPF conditions and maintained in a BALB/c background.

### Mouse experiments

Female BALB/c mice (5–6 weeks old, 18–20 g) were obtained from Shanghai SLAC Laboratory Animal Co., Ltd. (Shanghai, China). HA-knockdown transgenic mice were maintained in our animal facility. For virus infection, mice were anaesthetized and inoculated intranasally with 5 × 10^4^ PFU of WSN or PR8 virus. At the indicated time, mice were euthanized and the lungs, spleens and thymuses were removed for further analysis by hematoxylin and eosin (H&E) staining, plaque assay, and Western blotting. Influenza virus infection of mice was carried out under enhanced BSL-2 (BSL-2+) conditions.

### Histopathological analysis

Histopathological analysis was performed as described previously^32^. Mouse tissues were fixed in 4% paraformaldehyde and embedded in paraffin. Thereafter, 5 μm thick sections were cut from each block, affixed firmly to clean microscope slides, deparaffinized and stained with H&E using an H&E staining kit (Solarbio, Beijing, China). Sections were examined under an Olympus BH-2 microscope (Tokyo, Japan).

### Plaque assay

MDCK cells were infected with serial dilutions of the viruses. After an incubation period, cells were washed with PBS and overlaid with DMEM containing 1.5% low melting point agarose (Promega, Madison, WI) and 2 μg/ml TPCK (tolylsulfonyl phenylalanyl chloromethyl ketone)-treated trypsin (Sigma-Aldrich, St. Louis, MO). After 72 h of incubation at 37 °C, plaques were stained with 0.1605 mg/ml neutral red and number of plaques was counted.

### Examination of lung virus titers

Lungs harvested from sacrificed mice were homogenized in 1 ml of ice cold PBS and frozen at −80 °C for 14 h. Thawed samples were centrifuged at 2,000× gravity for 10 min, and the supernatants were titrated by plaque assay as described above.

### Statistical analysis

All data are presented as means ± standard errors [SE]. Survival curves were analyzed using the log-rank test (GraphPad Prism 5). Other statistical analysis was performed by Student’s *t* test. A level of P < 0.05 was considered statistically significant.

## Additional Information

**How to cite this article**: Wang, S. *et al*. Targeted disruption of influenza A virus hemagglutinin in genetically modified mice reduces viral replication and improves disease outcome. *Sci. Rep.* **6**, 23746; doi: 10.1038/srep23746 (2016).

## Supplementary Material

Supplementary Information

## References

[b1] Palese P. & Young J. F. Variation of influenza A, B, and C viruses. Science 215, 1468–1474 (1982). 7038875 10.1126/science.7038875

[b2] Jagger B. W. . An overlapping protein-coding region in influenza A virus segment 3 modulates the host response. Science 337, 199–204 (2012). 22745253 10.1126/science.1222213 PMC3552242

[b3] Palese P. Influenza: old and new threats. Nat. Med. 10, S82–87 (2004). 15577936 10.1038/nm1141

[b4] Horimoto T. & Kawaoka Y. Pandemic threat posed by avian influenza A viruses. Clin. Microbiol. Rev. 14, 129–149 (2001). 11148006 10.1128/CMR.14.1.129-149.2001 PMC88966

[b5] Guarnaccia T. . Antigenic drift of the pandemic 2009 A(H1N1) influenza virus in A ferret model. PLoS Pathog 9, e1003354 (2013). 23671418 10.1371/journal.ppat.1003354 PMC3649996

[b6] Ferguson N. M. . Strategies for mitigating an influenza pandemic. Nature 442, 448–452 (2006). 16642006 10.1038/nature04795 PMC7095311

[b7] Hota S. & McGeer A. Antivirals and the control of influenza outbreaks. Clin. Infect. Dis. 45, 1362–1368 (2007). 17968836 10.1086/522661

[b8] Subbarao K., Murphy B. R. & Fauci A. S. Development of effective vaccines against pandemic influenza. Immunity 24, 5–9 (2006). 16413916 10.1016/j.immuni.2005.12.005

[b9] Daly J. M., MacRae S., Newton J. R., Wattrang E. & Elton D. M. Equine influenza: a review of an unpredictable virus. Vet. J. 189, 7–14 (2011). 20685140 10.1016/j.tvjl.2010.06.026

[b10] De Clercq E. Antiviral agents active against influenza A viruses. Nat Rev Drug Discov 5, 1015–1025 (2006). 17139286 10.1038/nrd2175 PMC7097821

[b11] Boltz D. A., Aldridge J. R. Jr., Webster R. G. & Govorkova E. A. Drugs in development for influenza. Drugs 70, 1349–1362 (2010). 20614944 10.2165/11537960-000000000-00000 PMC5558450

[b12] Dong G. . Adamantane-resistant influenza a viruses in the world (1902–2013): frequency and distribution of M2 gene mutations. PLoS One 10, e0119115 (2015). 25768797 10.1371/journal.pone.0119115 PMC4358984

[b13] Nelson M. I., Simonsen L., Viboud C., Miller M. A. & Holmes E. C. The origin and global emergence of adamantane resistant A/H3N2 influenza viruses. Virology 388, 270–278 (2009). 19394063 10.1016/j.virol.2009.03.026 PMC2705899

[b14] Bloom J. D., Gong L. I. & Baltimore D. Permissive secondary mutations enable the evolution of influenza oseltamivir resistance. Science 328, 1272–1275 (2010). 20522774 10.1126/science.1187816 PMC2913718

[b15] Hay A. J. & Hayden F. G. Oseltamivir resistance during treatment of H7N9 infection. Lancet 381, 2230–2232 (2013). 23809549 10.1016/S0140-6736(13)61209-X

[b16] Samson M., Pizzorno A., Abed Y. & Boivin G. Influenza virus resistance to neuraminidase inhibitors. Antiviral Res. 98, 174–185 (2013). 23523943 10.1016/j.antiviral.2013.03.014

[b17] Moscona A. Medical management of influenza infection. Annu. Rev. Med. 59, 397–413 (2008). 17939760 10.1146/annurev.med.59.061506.213121

[b18] Meister G. & Tuschl T. Mechanisms of gene silencing by double-stranded RNA. Nature 431, 343–349 (2004). 15372041 10.1038/nature02873

[b19] Wilson R. C. & Doudna J. A. Molecular mechanisms of RNA interference. Annu Rev Biophys 42, 217–239 (2013). 23654304 10.1146/annurev-biophys-083012-130404 PMC5895182

[b20] Tompkins S. M., Lo C. Y., Tumpey T. M. & Epstein S. L. Protection against lethal influenza virus challenge by RNA interference *in vivo*. Proc. Natl. Acad. Sci. USA 101, 8682–8686 (2004). 15173583 10.1073/pnas.0402630101 PMC423255

[b21] Ge Q. . Inhibition of influenza virus production in virus-infected mice by RNA interference. Proc. Natl. Acad. Sci. USA 101, 8676–8681 (2004). 15173599 10.1073/pnas.0402486101 PMC423254

[b22] Ge Q. . RNA interference of influenza virus production by directly targeting mRNA for degradation and indirectly inhibiting all viral RNA transcription. Proc. Natl. Acad. Sci. USA 100, 2718–2723 (2003). 12594334 10.1073/pnas.0437841100 PMC151407

[b23] Wise T., Schafer D., Lowenthal J. & Doran T. The use of RNAi and transgenics to develop viral disease resistant livestock. Dev Biol (Basel) 132, 377–382 (2008). 18817330 10.1159/000317188

[b24] Jain B. . *In vitro* validation of self designed “universal human Influenza A siRNA”. Indian J. Exp. Biol. 53, 514–521 (2015). 26349314

[b25] Svancarova P., Svetlikova D. & Betakova T. Synergic and antagonistic effect of small hairpin RNAs targeting the NS gene of the influenza A virus in cells and mice. Virus Res. 195, 100–111 (2015). 25192613 10.1016/j.virusres.2014.08.004

[b26] Daniel-Carlier N. . Viral infection resistance conferred on mice by siRNA transgenesis. Transgenic Res. 22, 489–500 (2013). 22961198 10.1007/s11248-012-9649-4

[b27] Pengyan W. . Transgenic mouse model integrating siRNA targeting the foot and mouth disease virus. Antiviral Res. 87, 265–268 (2010). 20176056 10.1016/j.antiviral.2010.02.319

[b28] Lin L., Liu Q., Berube N., Detmer S. & Zhou Y. 5′-Triphosphate-short interfering RNA: potent inhibition of influenza A virus infection by gene silencing and RIG-I activation. J. Virol. 86, 10359–10369 (2012). 22787226 10.1128/JVI.00665-12 PMC3457308

[b29] Sui H. Y. . Small interfering RNA targeting m2 gene induces effective and long term inhibition of influenza A virus replication. PLoS One 4, e5671 (2009). 19479060 10.1371/journal.pone.0005671 PMC2682565

[b30] Perrimon N., Ni J. Q. & Perkins L. *In vivo* RNAi: today and tomorrow. Cold Spring Harb Perspect Biol 2, a003640 (2010). 20534712 10.1101/cshperspect.a003640 PMC2908776

[b31] Stevens J. . Structure and receptor specificity of the hemagglutinin from an H5N1 influenza virus. Science 312, 404–410 (2006). 16543414 10.1126/science.1124513

[b32] Fan K. . Role of Itk signalling in the interaction between influenza A virus and T-cells. J. Gen. Virol. 93, 987–997 (2012). 22302878 10.1099/vir.0.041228-0

[b33] Liu B. . Severe influenza A(H1N1)pdm09 infection induces thymic atrophy through activating innate CD8(+)CD44(hi) T cells by upregulating IFN-gamma. Cell Death Differ. 5, e1440 (2014). 10.1038/cddis.2014.323 PMC4649502 25275588

[b34] Yang S. . Cross-protective immunity against influenza A/H1N1 virus challenge in mice immunized with recombinant vaccine expressing HA gene of influenza A/H5N1 virus. Virol J 10, 291 (2013). 24053449 10.1186/1743-422X-10-291 PMC3848947

[b35] La Gruta N. L. & Turner S. J. T cell mediated immunity to influenza: mechanisms of viral control. Trends Immunol 35, 396–402 (2014). 25043801 10.1016/j.it.2014.06.004

[b36] Thomas P. G., Keating R., Hulse-Post D. J. & Doherty P. C. Cell-mediated protection in influenza infection. Emerging Infect. Dis. 12, 48–54 (2006). 16494717 10.3201/eid1201.051237 PMC3291410

[b37] Monto A. S. Vaccines and antiviral drugs in pandemic preparedness. Emerging Infect. Dis. 12, 55–60 (2006). 16494718 10.3201/eid1201.051068 PMC3291404

[b38] Lyall J. . Suppression of avian influenza transmission in genetically modified chickens. Science 331, 223–226 (2011). 21233391 10.1126/science.1198020

[b39] Grimm D. . Fatality in mice due to oversaturation of cellular microRNA/short hairpin RNA pathways. Nature 441, 537–541 (2006). 16724069 10.1038/nature04791

[b40] Martin J. N. . Lethal toxicity caused by expression of shRNA in the mouse striatum: implications for therapeutic design. Gene Ther. 18, 666–673 (2011). 21368900 10.1038/gt.2011.10 PMC3131434

[b41] McBride J. L. . Artificial miRNAs mitigate shRNA-mediated toxicity in the brain: implications for the therapeutic development of RNAi. Proc. Natl. Acad. Sci. USA 105, 5868–5873 (2008). 18398004 10.1073/pnas.0801775105 PMC2311380

[b42] Boudreau R. L., Martins I. & Davidson B. L. Artificial microRNAs as siRNA shuttles: improved safety as compared to shRNAs *in vitro* and *in vivo*. Mol Ther 17, 169–175 (2009). 19002161 10.1038/mt.2008.231 PMC2834985

[b43] Cao W., Hunter R., Strnatka D., McQueen C. A. & Erickson R. P. DNA constructs designed to produce short hairpin, interfering RNAs in transgenic mice sometimes show early lethality and an interferon response. J Appl Genet 46, 217–225 (2005). 15876690

[b44] Stevens J. . Structure of the uncleaved human H1 hemagglutinin from the extinct 1918 influenza virus. Science 303, 1866–01870 (2004). 14764887 10.1126/science.1093373

[b45] Dreyfus C., Ekiert D. C. & Wilson I. A. Structure of a classical broadly neutralizing stem antibody in complex with a pandemic H2 influenza virus hemagglutinin. J. Virol. 87, 7149–7154 (2013). 23552413 10.1128/JVI.02975-12 PMC3676097

[b46] Ge S. & Wang Z. An overview of influenza A virus receptors. Crit. Rev. Microbiol. 37, 157–165 (2011). 21438845 10.3109/1040841X.2010.536523

[b47] Gamblin S. J. . The structure and receptor binding properties of the 1918 influenza hemagglutinin. Science 303, 1838–1842 (2004). 14764886 10.1126/science.1093155

[b48] Leung H. S. . Entry of influenza A Virus with a alpha2,6-linked sialic acid binding preference requires host fibronectin. J. Virol. 86, 10704–10713 (2012). 22837202 10.1128/JVI.01166-12 PMC3457276

[b49] Ito T. . Molecular basis for the generation in pigs of influenza A viruses with pandemic potential. J. Virol. 72, 7367–7373 (1998). 9696833 10.1128/jvi.72.9.7367-7373.1998 PMC109961

[b50] Khiabanian H., Trifonov V. & Rabadan R. Reassortment patterns in Swine influenza viruses. PLoS One 4, e7366 (2009). 19809504 10.1371/journal.pone.0007366 PMC2752997

[b51] Wang S. . Influenza A virus-induced degradation of eukaryotic translation initiation factor 4B contributes to viral replication by suppressing IFITM3 protein expression. J. Virol. 88, 8375–8385 (2014). 24829357 10.1128/JVI.00126-14 PMC4135930

[b52] Ouyang J. . NRAV, a long noncoding RNA, modulates antiviral responses through suppression of interferon-stimulated gene transcription. Cell Host Microbe 16, 616–626 (2014). 25525793 10.1016/j.chom.2014.10.001 PMC7104942

[b53] Wang S. . Transport of influenza virus neuraminidase (NA) to host cell surface is regulated by ARHGAP21 and Cdc42 proteins. J. Biol. Chem. 287, 9804–9816 (2012). 22318733 10.1074/jbc.M111.312959 PMC3323004

[b54] Wei H. . Suppression of interferon lambda signaling by SOCS-1 results in their excessive production during influenza virus infection. PLoS Pathog 10, e1003845 (2014). 24391501 10.1371/journal.ppat.1003845 PMC3879354

[b55] Yang J. . eIF4B phosphorylation by pim kinases plays a critical role in cellular transformation by Abl oncogenes. Cancer Res. 73, 4898–4908 (2013). 23749639 10.1158/0008-5472.CAN-12-4277

