# Supplementary material for: Targeted disruption of influenza A virus hemagglutinin in genetically modified mice reduces viral replication and improves disease outcome
Source: Sci Rep. 2016 Apr 1;6:23746. doi: 10.1038/srep23746 (PMC4817130; doi:10.1038/srep23746)
Supplement: Supplementary Information [file srep23746-s1.pdf]

Supplementary Information

Targeted disruption of influenza A virus hemagglutinin in genetically modified mice reduces viral replication and improves disease outcome

Song Wang[1#], Chao Chen[1#], Zhou Yang[1], Xiaojuan Chi[1], Jing Zhang[2], and Ji-Long Chen[1,2*]

[1] College of Animal Sciences, Fujian Agriculture and Forestry University, Fuzhou 350002, Fujian, China

[2] Institute of Microbiology, Chinese Academy of Sciences (CAS), Beijing 100101, China

*Corresponding author: Ji-Long Chen. Email: chenjl@im.ac.cn; Tel: 086-10-64807300; Fax: 086-10-64807980

[#]These authors contributed equally to this work.

Supplementary Figure S1

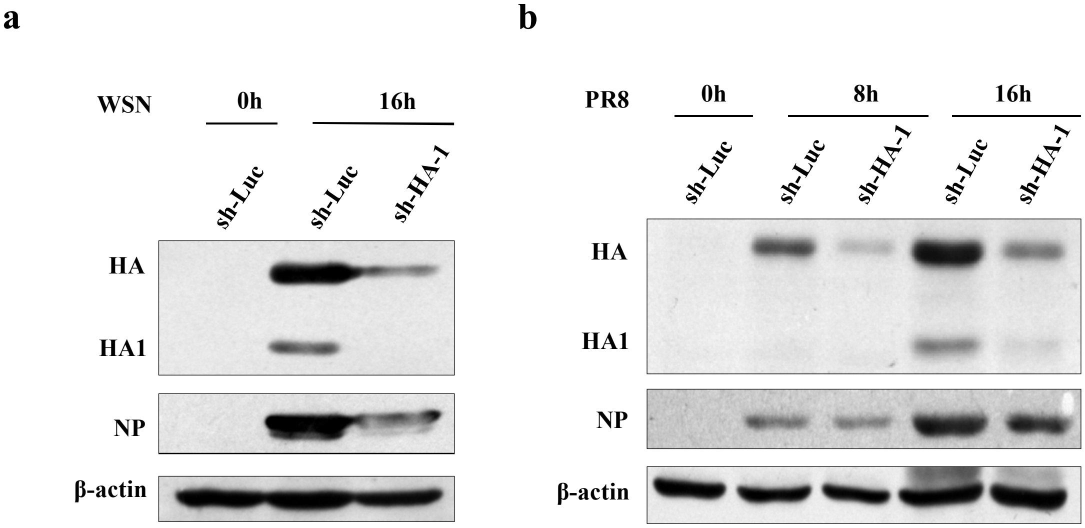

Figure S1. HA and NP expressions in HA knockdown A549 cells. (a, b) A549 cells stably expressing shRNAs targeting either HA (sh-HA-1) or luciferase control (sh-Luc) were infected with WSN virus (a) or PR8 virus (b) at an MOI of 0.5 and harvested at the indicated times, followed by Western blotting using the indicated antibodies.

Supplementary Figure S2

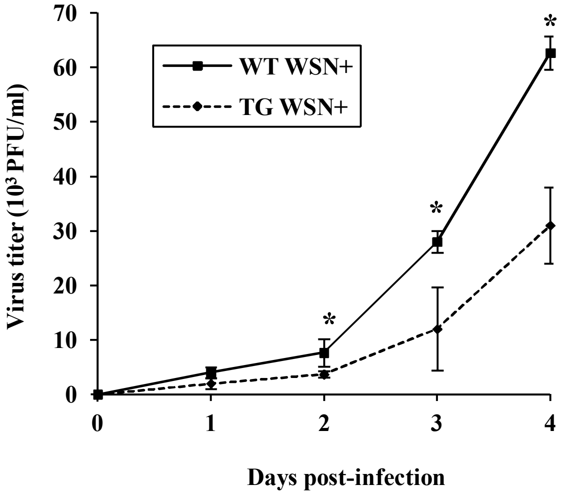

Figure S2. Viral loads in the lungs of WT and TG mice. WT and TG mice intranasally infected with WSN virus were sacrificed at the indicated times, and the viral loads in the mice lungs were measured by plaque assay. *P= 0.03709 (WSN infection for 2 days); *P=0.019053 (WSN infection for 3 days); P= 0.015885 (WSN infection for 4 days).

Supplementary Figure S3

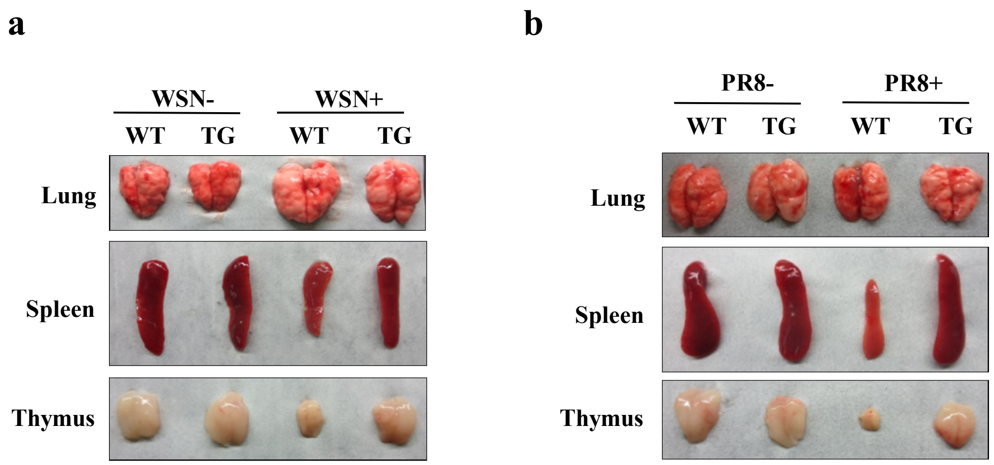

Figure S3. Representative lung, spleen and thymus images of WT and TG mice infected with WSN or PR8 virus. WT and TG mice were mock infected or infected intranasally with WSN or PR8 virus for 4 days. Then mice were sacrificed, and the lungs, spleens and thymuses were collected. (a) and (b) are representative images from three independent experiments.

Supplementary Figure S4

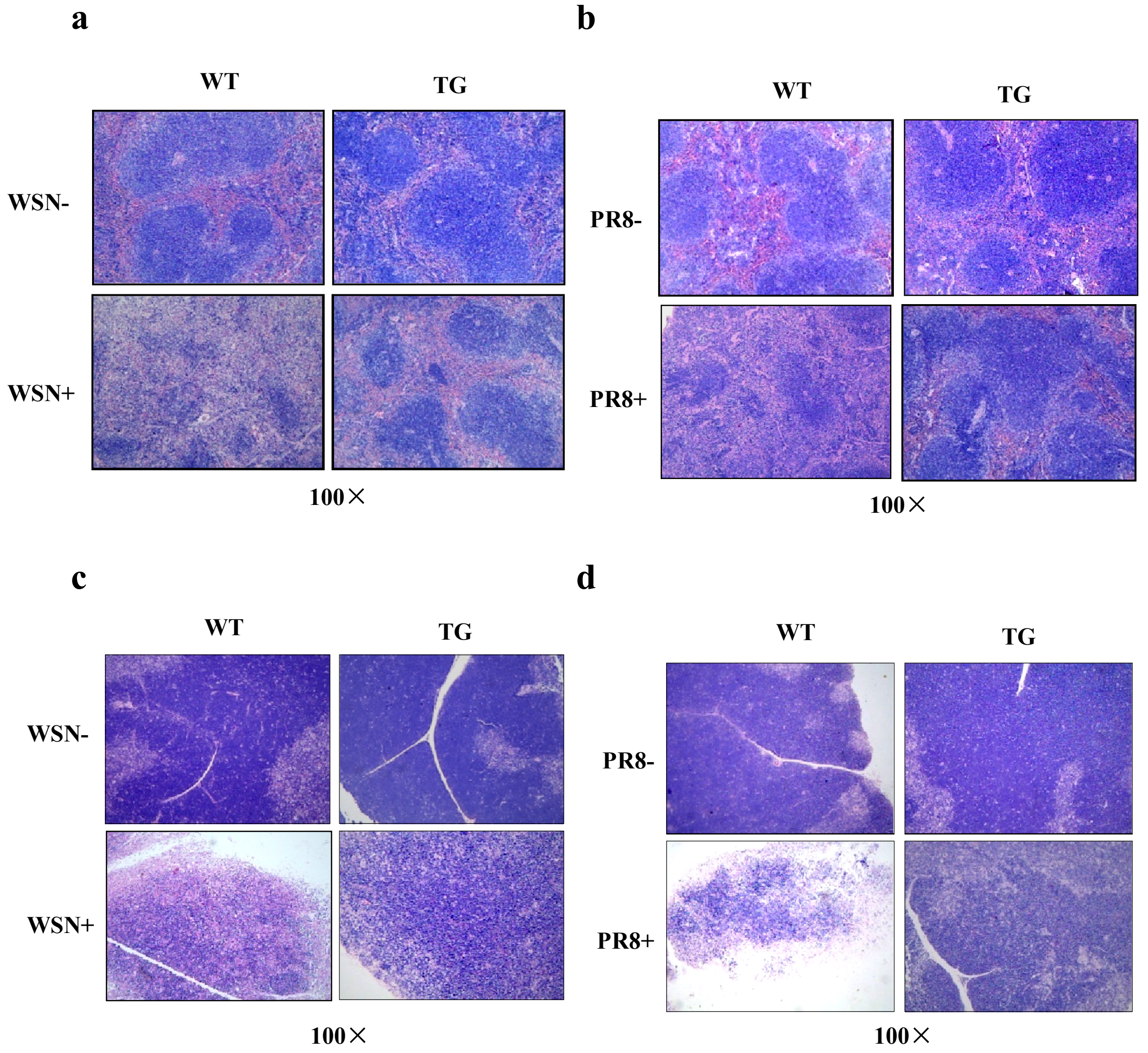

Figure S4. The pathological changes in spleens and thymuses of WT and TG mice after influenza virus infection. (a, b) WT and TG mice were intranasally infected with WSN (a) or PR8 (b) virus. Shown are representative micrographs (magnification, ×100) of the mouse spleens stained with hematoxylin and eosin (HE). (c, d) WT and TG mice were intranasally infected with WSN (c) or PR8 (d) virus as described in (a) and (b). Shown are representative micrographs (magnification, ×100) of the mouse thymuses stained with HE.

Supplementary Figure S5

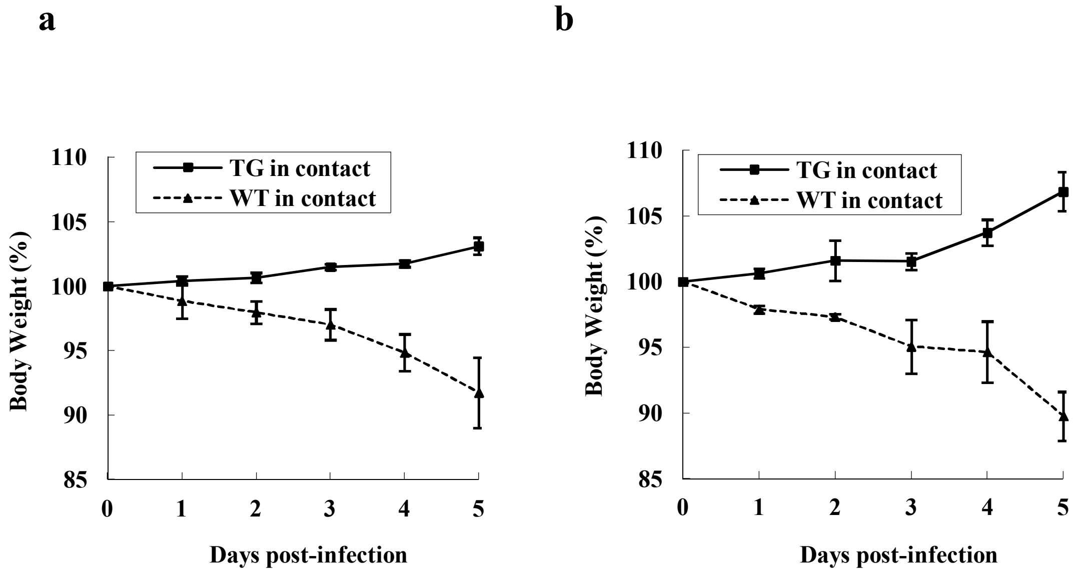

Figure S5. The onward transmission of influenza virus in contact with influenza virus infected WT or TG mice. (a) Two groups of six unchallenged WT mice were housed with three WSN-infected WT mice (WT in contact) or TG mice (TG in contact) for 5 days. Shown is the average body weight changes of groups of six unchallenged WT mice. (b) Two groups of six unchallenged WT mice were housed with three PR8-infected WT mice (WT in contact) or TG mice (TG in contact) for 5 days. Shown is the average body weight changes of groups of six unchallenged WT mice.

Supplementary Table S1

Representative influenza A virus strains sharing the conserved sequences in the globular region of hemagglutinin located in HA1 domain.

Influenza virus strains	Sequence
A/swine/Iowa/A02077564/2015(H1N2)	AGGTCAAACAG[#]GGAGGATGAACTATTACTGGACAATAGT
A/Oklahoma/06/2014(H1N1)	GGATCAAGAAGGGAGGATGAACTATTACTGGACACTAGT
A/LongYan/SWL35/2013(H1N1)	AGATCAAGAAGGGAGGATGAACTATTACTGGACACTAGT
A/swine/Mie/R02/2012(H1N2)	AGATCAGGCAGGGAGGATGAACTATTACTGGACATTGAT
A/Beijing/38/2012(H1N1)	GGATCAAGAAGGGAGGATGAACTATTACTGGACACTAGT
A/swine/Tochigi/2/2011(H1N2)	AGATCAGGCAGGGAGGATGAACTATTACTGGACATTGAT
A/Hawaii/05/2011(H1N1)	GGATCAAGAAGGGAGGATGAACTATTACTGGACACTAGT
A/swine/Korea/4941/2010(H1N2)	GGACCAAGAAGGGAGGATGAACTATTACTGGACACTAGT
A/swine/Manitoba/02947/2010(H1N1)	AGATCAAGCAGGGAGGATGAACTATTACTGGACATTAGT
A/Kansas/21/2009(H1N1)	GGATCAAGAAGGGAGGATGAACTATTACTGGACACTAGT
A/swine/4/Mexico/2009(H1N1)	GRRTCRAGAAGGGAGGATGAACTATTACTGGACACTAGT
A/Hangzhou/4/2009(H1N1)	GGATCAAGAAGGGAGGATGAACTATTACTGGACACTAGT
A/swine/Saskatchewan/01974/2008(H1N1)	AGATCAAGCAGGGAGGATGAACTATTACTGGACATTAGT
A/swine/Manitoba/01781/2007(H1N1)	AGATCAAGCAGGGAGGATGAACTATTACTGGACATTAGT
A/swine/Alberta/SG1415/2006(H1N1)	AGATCAAGCAGGGAGGATGAACTATTACTGGACATTAGT
A/swine/Hong Kong/729/2005(H1N2)	GGACCAAGCAGGGAGGATGAACTATTACTGGACACTAGT
A/swine/Niigata/729/2004(H1N2)	AAATCAAACAGGGAGGATGAACTATTACTGGACACTAAT
A/swine/Changhua/199-3/2000(H1N1)	AGGTCAGGCAGGGAGGATGAACTATTACTGGACATTACT
A/swine/Chiba/1/1991(H1N2)	AGGTCAAGCAGGGAGGATGAACTATTACTGGACACTAAT
A/swine/Ehime/1/1980(H1N2)	AGGTCAAGCAGGGAGGATGAACTATTACTGGACACTAAT
A/swine/Tennessee/9/1978(H1N1)	AGGTCAAGCAGGGAGGATGAACTATTACTGGACACTAAT
A/swine/Hong Kong/26/1977(H1N1)	AGGTCAAGCAGGGAGGATGAACTATTACTGGACACTAAT
A/swine/Wisconsin/11/1976(H1N1)	AGGTCAAGCAGGGAGGATGAACTATTACTGGACACTAAT
A/New Jersey/8/1976(H1N1)	AGGTCAAGCAGGGAGGATGAACTATTACTGGACACTAAT
A/swine/Wisconsin/1/1968(H1N1)	AGGTCAGGCAGGGAGGATGAACTATTACTGGACATTAAT
A/swine/Wisconsin/1/1957(H1N1)	AGGTCAAGCAGGGAGGATGAACTATTACTGGACATTAAT
A/Hemsbury/1948(H1N1)	AGATCAAGCAGGGAGGATGAACTATTACTGGACCCTGCT
A/Puerto Rico/8/1934(H1N1)	AGATCAAGCTGGGAGGATGAACTATTACTGGACCTTGCT
A/WSN/1933(H1N1)	AGATCAACATGGGAGGATGAACTATTACTGGACCTTGCT
A/swine/JY2/1931(H1N1)	AGGTCAGGCAGGGAGGATGAACTATTACTGGACATTACT
A/South Carolina/1/1918 (H1N1)	AGATCAAGCTGGGAGGATGAACTATTACTGGACATTACT
A/duck/Hong Kong/91/1976(H9N2)	TGGTCAGCAGGGGAGGATT[*]GACTATTATTGGTCGGTACT
A/duck/Malaysia/2001(H9N2)	TGGCCAGCAGGGGAGGATTGACTATTATTGGTCAGTACT
A/duck/Jiangxi/3292/2009(H7N9)	TGGCCAATCTGGAAGGATTGACTTTCATTGGCTGATGCT
A/wild duck/Korea/MHC39-26/2011(H7N9)	TGGCCAATCTGGAAGGATTGACTTTCATTGGCTGATACT

[#] The nucleotides in red font are the same with the target sequences.

[*] The nucleotides in blue font are different from the target sequences.

Supplementary Table S2

Representative influenza A virus strains sharing the conserved sequences in the stem region of hemagglutinin located in HA2 domain.

Influenza virus strains	Sequence
A/Xinjiang/1/2009(H5N1)	ATAATGAATGCATGGAAAGTGTAAGAAATGGAACGTATG
A/Vietnam/UT31641-2/2008(H5N1)	ATAATGAATGCATGGAAAGTGTAAGAAATGGAACGTATG
A/quail/Mingalardone/866/2007(H5N1)	ATAATGAATGCATGGAAAGTGTAAGAAATGGAACGTATG
A/chicken/Bac Giang/07-74/2007(H5N1)	ATAATGAATGCATGGAAAGTGTAAGAAATGGAACGTATG
A/duck/New York/484680/2007(H5N2)	ACAATGAGTGCATGGAAAGTGTAAGAAATGGAACGTATG
A/parrot/Guangdong/258/2004(H5N2)	ACAATGAATGCATGGAAAGTGTAAGAAATGGAACGTATG
A/chicken/Texas/298313/2004(H5N2)	ACAATGAATGCATGGAAAGTGTAAGAAATGGAACATATA
A/chicken/Guatemala/270475-4/2003(H5N2)	ACAATGAATGCATGGAAAGTGTAAGAAATGGAACTTATG
A/swine/Gent/177/2002(H1N2)	ACAATGAGTGCATGGAAAGTGTAAGAAATGGAACTTATG
A/duck/NY/191255-79/2002(H5N2)	ACAATGAGTGCATGGAAAGTGTAAGAAATGGAACGTATG
A/mallard/Maryland/786/2002(H5N2)	ACAATGAATGCATGGAAAGTGTAAGAAATGGAACGTATG
A/chicken/TX/167280-4/2002(H5N3)	ACAATGAATGCATGGAAAGTGTAAGAAATGGAACATATA
A/duck/NY/191255-59/2002(H5N8)	ACAATGAGTGCATGGAAAGTGTAAGAAATGGAACGTATG
A/avian/New York/Sg-00372/2001(H5N2)	ACAATGAGTGCATGGAAAGTGTAAGAAATGGAACGTATG
A/mallard/Maryland/252/2001(H5N2)	ACAATGAATGCATGGAAAGTGTAAGAAATGGAACGTATG
A/Avian/NY/53726/2000(H5N2)	ACAATGAATGCATGGAAAGTGTAAGAAATGGAACGTATG
A/chukar/MN/14591-7/1998(H5N2)	ACAATGAATGCATGGAAAGTGTAAGAAATGGAACGTATG
A/mallard/MN/133/1998(H5N2)	ACAATGAATGCATGGAAAGTGTAAGAAATGGAACGTATG
A/Mongolia/111/1991 (H1N1)	ACAATGAATGCATGGAAAGTGTAAGAAATGGGACTTATG
A/Tokyo/3/1967(H1)	ACAATAAATGCATGGAAAGTGTAAGAAATGGGACTTATG
A/BH/JY2/1935(H1N1)	ACAATGAATGCATGGAAAGTGTAAGAAATGGGACTTATG
A/Puerto Rico/8/1934(H1N1)	ACAATGAATGCATGGAAAGTGTAAGAAATGGGACTTATG
A/WSN/1933(H1N1)	ACAATGAATGCATGGAAAGTGTAAGAAATGGGACTTATG
A/South Carolina/1/1918 (H1N1)	ACGATGCATGCATGGAAAGTGTAAGAAATGGGACTTATG
A/chicken/Bangladesh/23527/2014(H9N2)	ATGACCAATGCATGGAAACAATTCGAAATGGGACCTATA
A/chicken/Heilongjiang/u/1998(H9N2)	ATGATCAATGCATGGAAGCAATCAGAAATGGAACCTACG
A/Kansas/04/2015(H3N2)	ACAATGCCTGCATAGGATCAATAAGAAATGGAACTTATG
A/Hong Kong/485197/2014(H3N2)	ACAATGCCTGCATAGGATCAATAAGAAATGGAACTTATG
